# Community perceptions of pre-eclampsia and eclampsia in Ogun State, Nigeria: a qualitative study

**DOI:** 10.1186/s12978-016-0134-z

**Published:** 2016-06-08

**Authors:** David O Akeju, Marianne Vidler, Olufemi T Oladapo, Diane Sawchuck, Rahat Qureshi, Peter von Dadelszen, Olalekan O Adetoro, Olukayode A Dada, the CLIP Nigeria Feasibility Working Group

**Affiliations:** Department of Sociology, University of Lagos, Lagos, Nigeria; Department of Obstetrics and Gynaecology and the Child and Family Research Unit, University of British Columbia, Vancouver, British Columbia Canada; UNDP/UNFPA/UNICEF/WHO/World Bank Special Programme of Research, Development and Research Training in Human Reproduction (HRP), Department of Reproductive Health and Research, World Health Organization, Geneva, Switzerland; Department of Obstetrics and Gynaecology, Aga Khan University, Aga Khan, Pakistan; Department of Obstetrics and Gynaecology, Olabisi Onabanjo University, Sagamu, Ogun State Nigeria; Centre for Research in Reproductive Health, Sagamu, Ogun State Nigeria

**Keywords:** Community, Perception, Pre-eclampsia, Eclampsia, Nigeria, Hypertension, Seizures, Attitudes

## Abstract

**Background:**

Pre-eclampsia is a complication of pregnancy responsible for high rates of morbidity and mortality, particularly in sub-Saharan Africa. When undetected or poorly managed, it may progress to eclampsia which further worsens the prognosis. While most studies examining pre-eclampsia have used a bio-medical model, this study recognizes the role of the socio-cultural environment, in order to understand perceptions of pre-eclampsia within the community.

**Methods:**

The study was conducted in Ogun State, Nigeria in 2011–2012. Data were obtained through twenty-eight focus group discussions; seven with pregnant women (*N* = 80), eight with new mothers (*N* = 95), three with male decision-makers (*N* = 35), six with community leaders (*N* = 68), and three with traditional birth attendants (*N* = 36). Interviews were also conducted with the heads of the local traditional birth attendants (*N* = 4) and with community leaders (*N* = 5). Data were transcribed verbatim and analysed in NVivo 10 software.

**Results:**

There was no terminology reportedly used for pre-eclampsia in the native language - *Yoruba*; however, hypertension has several terms independent of pregnancy status. Generally, ‘*gìrì âlábôyún’* describes seizures specific to pregnancy. The cause of hypertension in pregnancy was thought to be due to depressive thoughts as a result of marital conflict and financial worries, while seizures in pregnancy were perceived to result from prolonged exposure to cold. There seemed to be no traditional treatment for hypertension. However for seizures the use of herbs, concoctions, incisions, and topical application of black soap were widespread.

**Conclusion:**

This study illustrates that knowledge of pre-eclampsia and eclampsia are limited amongst communities of Ogun State, Nigeria. Findings reveal that pre-eclampsia was perceived as a stress-induced condition, while eclampsia was perceived as a product of prolonged exposure to cold. Thus, heat-related local medicines and herbal concoctions were the treatment options. Perceptions anchored on cultural values and lack of adequate and focused public health awareness is a major constraint to knowledge of the aetiology and treatment of the conditions. A holistic approach is recommended for sensitization at the community level and the need to change the community perceptions of pre-eclampsia remains a challenge.

**Trial Registration:**

NCT01911494.

**Electronic supplementary material:**

The online version of this article (doi:10.1186/s12978-016-0134-z) contains supplementary material, which is available to authorized users.

## Background

Pre-eclampsia is one of the most common complications of pregnancy and continues to be a leading cause of death and disability globally [1]. Pre-eclampsia is characterized by new onset of hypertension and proteinuria after 20 weeks gestation [2]. It may progress to eclampsia; a potentially lethal complication characterized by convulsions requiring an emergency response [3]. The World Health Organization estimates that 14 % of all maternal deaths result from the hypertensive disorders of pregnancy (HDP); it is also associated with a high risk of newborn death [1]. Nigeria, has one of the highest maternal mortality ratios ranging from 496 to 560 per 100,000 live births [4, 5], as well as a high prevalence of pre-eclampsia and eclampsia of between 2 % to 16.7 % [6].

Most studies focussing on pre-eclampsia and eclampsia have used a bio-medical model to examine causative factors, prevention and treatment without much attention to local perceptions. This study takes an alternate approach by adopting a perspective that recognizes an interaction of various components of the socio-cultural environment that influence community perceptions [7]. The aetiology of pre-eclampsia remains a mystery; the cause and disease pathways are not fully understood [8]. There is a gap in knowledge regarding the causes among communities in Northern Nigeria where pre-eclampsia is believed to be caused by *Iska* (spirits) [9]. Similarly, Nigerian men have reported that maternal deaths are caused by supernatural spirits, social and economic factors, or poor quality health care services [10]. Some Nigerians did not recognize the signs of pre-eclampsia; however, some women recognized eclampsia and hypertension as potential causes of maternal mortality [11, 12].

The aim of this study was to identify community perceptions of pre-eclampsia and eclampsia in Ogun State, Nigeria. These community understandings were described by the use of local terms, perceived causes, prevention strategies, known outcomes and traditional treatments.

## Methods

### Description of study sites

**Fig. 1:**
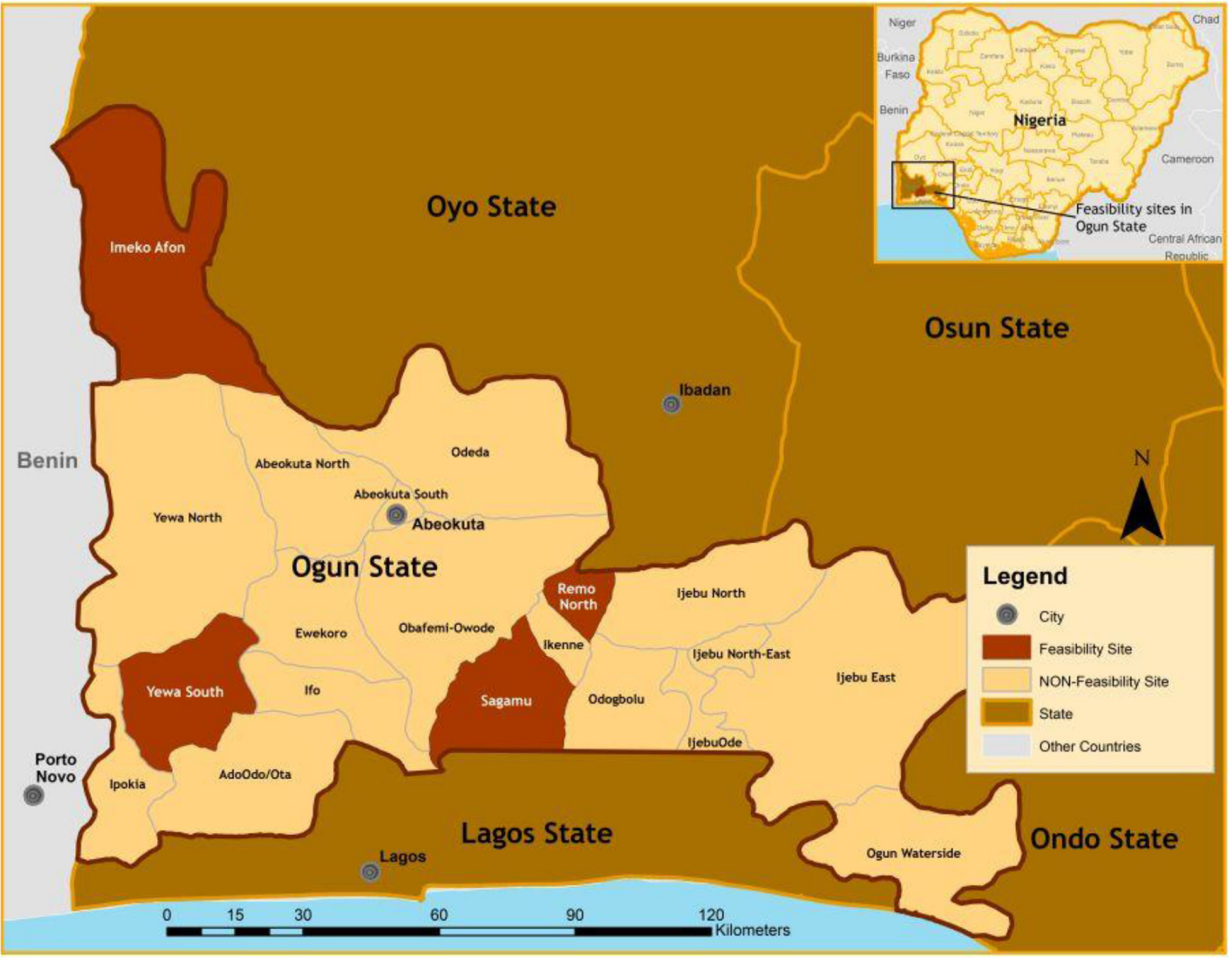
Map of study sites

The study was undertaken in Nigeria, the country with the largest population in Africa and the 14^th^ largest in land mass [4]. More specifically, it was conducted in four (Ogijo, Yewa South, Imeko-Afon and Remo North) Local Government Areas (LGAs) in Ogun State, located in south-western Nigeria. The predominant ethnic group is Yoruba. The health status of the region is reflective of that of most of south-western Nigeria [4]. There are high rates of poverty, fertility and mortality [4]. See Table 1 for country and cluster characteristics, see Fig. 1 for a map of study areas.

**Table 1:** Study site characteristics

Nigeria characteristics	
Population	159,288,426
Size (Km^2^)	923, 768
Number of states	36
Number of geopolitical zones	6
Predominant language	Yoruba, Igbo, and Hausa
Predominant religions	Christianity and Islam
Ogun State characteristics	
Population	4,000,000
Size (Km^2^)	16,409
Number of local government areas	20
Predominant language	Yoruba
Predominant religion	Christianity
Local Government Area characteristics	
Cumulative population	469,271
Cumulative size (Km^2^)	1657
Number of study areas	4/40

### Data collection

Data were collected as part of a larger community-based assessment of the acceptability of community-based treatment for pre-eclampsia and eclampsia in Nigeria: a detailed description of these methods is published elsewhere [13]. Focus group discussions and in-depth interviews were conducted from 2011-to-2012.

Eight focus groups were held with pregnant women (*N* = 94), eight with new mothers (*N* = 95), four with male decision-makers (*N* = 47), five with community leaders (religious and representatives of traditional rulers and political groups) (*N* = 56), and three with traditional birth attendants (TBAs) (*N* = 36). In addition, interviews were conducted with the heads of the traditional birth attendants (*N* = 4) and with community leaders (*N* = 5).

### Data analysis

**Fig. 2:**
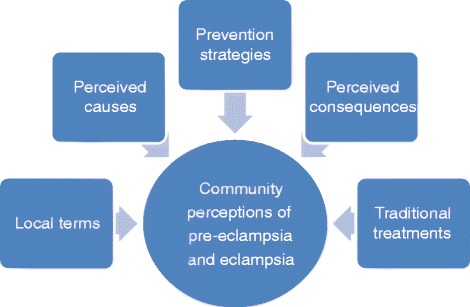
Thematic categories used in analysis

All in-depth interviews and focus group discussions were transcribed and translated from local language (Yoruba) to English. These translations were reviewed by study investigators to ensure accuracy, and validated with field notes. Data were analysed using QSR NVivo version 10 software. The analytical framework and coding structure were developed by the study principal investigators (FO, OA) and data analyst (MV); however, for consistency, all coding was performed by one individual (MV). In addition, the nodal structure created and used for coding was subjected to a thorough review process by five other authors prior to the commencement of the coding exercise. The majority of themes were identified a priori to represent community perceptions of pre-eclampsia; however, a small number of themes emerged from the data through the coding process and were included at that time (Fig. 2).

The Health Research and Ethics Committee of Olabisi Onabanjo University Teaching Hospital, Sagamu, Nigeria (OOUTH/DA/326/431), and the Clinical Research Ethics Board of the University of British Columbia, Vancouver, Canada (H12-00132), approved the study.

## Results

Demographic characteristics were collected during focus groups discussions on all 323 participants; for details of participants see Tables 2. Nearly all participants were married; there were very few exceptions to this. Participants were of Islamic, Christian, and local traditional faiths; however, Christianity predominated. Most participants had at least one child with several having up to 7 children. Educational attainment was highest in one cluster (Ogijo); this may be explained by its proximity to an urban centre (Tables 2 and 3).

**Table 2:** Focus group discussion characteristics

#	*N* participants	Region	Age (yr) median [range]	Religion	*N* children median [range]	% Married
				1 Islam		
				2 Christian		
				3 Traditional religion		
Community leaders						
1	12	Yewa South	52 [27, 70]	1 = (*N* = 6)	5 [0, 6]	100 %
				2 = (*N* = 6)		
2	10	Remo North	44 [43, 77]	1 = (*N* = 3)	*Not known*	100 %
				2 = (*N* = 5)		
				3 = (*N* = 2)		
3	12	Remo North	58 [30, 85]	*Not known*	*Not known*	100 %
4	10	Ogijo	50 [26, 71]	1 = (*N* = 5)	6 [1, 9]	100 %
				2 = (*N* = 5)		
5	12	Ogijo	57 [45, 72]	1 = (*N* = 3)	8 [4, 10]	100 %
				2 = (*N* = 6)		
				3 = (*N* = 3)		
6	12	Imeko-Afon	45 [20, 55]	1 = (*N* = 6)	3 [0,10]	100 %
				2 = (*N* = 6)		
Male decision-makers						
1	12	Yewa South	38 [27, 49]	1 = (*N* = 2)	3 [1, 5]	100 %
				2 = (*N* = 10)		
2	11	Remo North	40 [35, 62]	1 = (*N* = 4)	3 [0, 9]	100 %
				2 = (*N* = 6)		
				3 = (*N* = 1)		
3	12	Imeko-Afon	51 [40, 60]	1 = (*N* = 11)	6 [4, 10]	100 %
				2 = (*N* = 1)		
New mothers						
1	12	Yewa South	27 [20, 42]	1 = (*N* = 6)	3 [1, 5]	100 %
				2 = (*N* = 6)		
2	12	Yewa South	31 [20, 42]	1 = (*N* = 4)	2 [1, 3]	100 %
				2 = (*N* = 8)		
3	12	Remo North	29 [21, 39]	1 = (*N* = 3)	4 [1, 6]	100 %
				2 = (*N* = 9)		
4	12	Remo North	28 [21, 34]	1 = (*N* = 5)	2 [1, 4]	100 %
				2 = (*N* = 7)		
5	12	Ogijo	31 [26, 43]	1 = (*N* = 4)	3 [1, 4]	92 %
				2 = (*N* = 8)		
6	12	Ogijo	29 [22, 38]	1 = (*N* = 1)	2 [1, 5]	100 %
				2 = (*N* = 11)		
7	12	Imeko-Afon	30 [16, 36]	1 = (*N* = 5)	3 [1, 5]	100 %
				2 = (*N* = 7)		
8	11	Imeko-Afon	30 [18, 36]	1 = (*N* = 6)	3 [1, 6]	100 %
				2 = (*N* = 5)		
Pregnant women						
1	12	Yewa South	26 [20, 33]	1 = (*N* = 3)	1 [0, 4]	100 %
				2 = (*N* = 9)		
2	12	Yewa South	26 [20, 39]	1 = (*N* = 4)	3 [1, 3]	100 %
				2 = (*N* = 8)		
3	12	Remo North	30 [20, 36]	1 = (*N* = 1)	1 [1, 3]	100 %
				2 = (*N* = 11)		
4	12	Remo North	32 [23, 40]	1 = (*N* = 5)	3 [1, 5]	100 %
				2 = (*N* = 7)		
5	9	Ogijo	27 [19, 34]	1 = (*N* = 6)	1 [0, 2]	100 %
				2 = (*N* = 3)		
6	10	Imeko-Afon	22 [19, 26]	1 = (*N* = 7)	1 [0, 4]	100 %
				2 = (*N* = 3)		
7	12	Imeko-Afon	25 [20, 30]	1 = (*N* = 2)	2 [0, 4]	100 %
				2 = (*N* = 10)		
Traditional birth attendants						
1	12	Yewa South	44 [32, 65]	1 = (*N* = 7)	3 [1, 4]	100 %
				1 = (*N* = 5)		
2	12	Remo North	50 [41, 77]	1 = (*N* = 1)	5 [3, 5]	100 %
				2 = (*N* = 8)		
				3 = (*N* = 2)		
3	12	Ogijo	40 [25, 50]	1 = (*N* = 5)	4 [0, 5]	83 %
				2 = (*N* = 6)

**Table 3:** Interview characteristics

#	Stakeholder group	Cluster
1	Head of traditional birth attendants	Sagamu
2	Head of traditional birth attendants	Yewa South
3	Head of traditional birth attendants	Imeko-Afon
4	Head of traditional birth attendants	Remo North
5	Community leader	Imeko-Afon
6	Male community leaders	Imeko-Afon
7	Women community leaders	Sagamu
8	Women community leaders	Imeko-Afon
9	Women community leaders	Remo North

### Local terms for pre-eclampsia and eclampsia

Based on the descriptions provided by respondents, pre-eclampsia and eclampsia appeared to be a common occurrence in these communities. Although, hypertension and convulsions had few local names that are independent of pregnancy status, there were no local terms in Yoruba for pre-eclampsia.

Hypertension in pregnancy was commonly referred to as “*èjè rírû*” or “*ìfúnpá gígâ*”. The term “*ìfúnpá gígâ”* in Yoruba, is a combination of two words: *“ìfúnpá”* signifies the process of strapping the arm during a blood pressure measurement and “*gígâ*” refers to something “elevated” or “high”. Thus, “*ìfúnpá gígâ*” as a local term was used to describe hypertension. On the other hand, *“èjè ríru”* provides a succinct description of hypertension; it literally means “*stormy blood”.* This term captures the local perception that hypertension is comparable to a storm within the body.

A number of participants used local terms for general convulsions or convulsions in children to describe eclampsia: “*gìrì”* and *“àìsàn îlè tútù”*. “*Gìrì”* is a common local term used to describe the temporary jerking of the body. “*Àìsàn îlè tútù”* is used to represent seizures, which translates to ‘*cold-ground illness’*; this indicated the perceived relationship between exposure to cold and seizures. Though these terms imply general convulsions, many also reported the use of ‘*gìrì âlábôyún’* which is specific to pregnancy and represents eclampsia (See Table 4 for a list of these local names).

**Table 4:** Local terms for pre-eclampsia and eclampsia

Local terms for pre-eclampsia		
Ìfúnpá gígâ	Èjè rírû (stormy blood)	
Local terms for eclampsia		
Gìrì âlábôyún (pregnancy-related seizure)	Gìrì (Seizure)	Gìrì àgbàlâgbà (Seizure in adults)
Gìrì ipa (stubborn seizure)	Gìrì înú ôyún (pregnancy-related seizure)	Òyì ôrí (dizziness in the head)
Îpá ná (Hot seizure)	Îlè tútù (cold ground)	Òyì ôjú (dizziness in the eyes)

### Perceived causes of pre-eclampsia and eclampsia

There was a consensus amongst the community regarding what was believed to cause hypertension in pregnancy; most often depressive thoughts and stress were described as the origin.

*“If the pregnant woman is having depressive thoughts, if she encounters something that is beyond her, and she begins to worry about the issue, a thing like that could cause high blood pressure.” [Pregnant Woman]*

The root of these depressive thoughts was most often related to marital or financial worries. This marital conflict included abandonment, teenage pregnancies, unfaithful partners and lack of adequate care by the husband.

*“It is caused by their husband’s bad behaviour, because a lot of men want their wives and not her pregnancy. Some husbands would stop taking care of their wives when they become pregnant.” [Community Leader]*

Depressive thoughts were also believed to be associated with a lack of rest which could cause hypertension. Most respondents in this study did not believe that hypertension could have a spiritual origin; however a small number maintained this belief.

Although a number of possible causes for eclampsia emerged from focus group discussions and in-depth interviews, the most common were the influences of cold, heredity, diet, and depressive thoughts or stress. There was a strong perception that taking cold food, or drinks during pregnancy can lead to convulsions, as well as the exposure to cold weather. This was by far the most common explanation of convulsions in pregnancy in the community. A pregnant woman demonstrates this belief in the following quote:

*“There is a belief that if a pregnant woman frequently sleeps on a cold floor, it could cause convulsion, or if the body is exposed to too much breeze […] and also if there are excessive depressive thoughts…it can lead to convulsion.” [Pregnant Woman]*

Another explanation was that convulsions during pregnancy could be hereditary.

*“Something that I have noticed about ‘gìrì’ is that some things are hereditary. There are some things that people would say similar thing happened to the father or mother at a time. So things like this would have become hereditary and if this is not treated early it will run from two to three generations and it would become a family problem.” [Community Leader]*

Similar to the causes of hypertension, development of seizures in pregnancy were seen to be caused by depressive thoughts and stress (See Table 5 for a list of causes mentioned). One male decision-maker shared his view about how depressive thoughts could lead to convulsion:

*“If she’s under too much stress, she might not sleep and it gets to the point that she finds it difficult to sleep and the health care workers begin to monitor her blood pressure, a thing like this could cause convulsion for an adult and it’s the same predicament for a pregnant woman” [Male Decision-Maker]*

**Table 5:** Perceived causes of pre-eclampsia and eclampsia

Perceived causes of pre-eclampsia	
Depressive thoughts	
Hereditary	
Stress	
Perceived causes of eclampsia	
Prolonged exposure to cold	
Hypertension	
Anaemia	
Malaria	
Urinary tract infections	
Diabetes	
Oedema	
Lack or loss of blood	
Pre-existing hypertension	
Taking cold food or drinks	
Sleeping on a cold floor	

Similarly, a number of respondents perceived the role of diet to be important in the development of seizures. Poor diet was rarely stated as an independent contributor, but rather a co-factor with marital problems or financial constraints. Apart from the causes mentioned above, the medical conditions thought to be related to eclampsia were anaemia, malaria, urinary tract infections, diabetes, infections, oedema, pre-existing hypertension, and the lack or loss of blood. The possibility of a spiritual origin for convulsions in pregnancy was widely discredited by the community. Some participants showed comprehension that high blood pressure is a cause of convulsions; however, most did not associate the two conditions.

*“What causes convulsion is like the other participant explained earlier, if a pregnant woman should have high blood pressure there would be a substance in her urine, there’s a way they detect the substance, also if her legs are swollen…blood shortage could cause swollen legs for the pregnant woman, lack of blood in her system could make her legs to swell. If a woman should stress herself too much during pregnancy a thing like this could make her develop high blood pressure, all these factors together would cause convulsion for the pregnant woman and the condition would be out of control. [Community Leader]*

### Prevention strategies for pre-eclampsia and eclampsia

Much of the preventive practices mentioned were related to pre-eclampsia and centred on the type and quality of care women received during pregnancy. There were a few mentions of local or traditional practices for prevention of pre-eclampsia aside from dietary suggestions. The role of men in the prevention of pre-eclampsia was widely emphasized, particularly the importance of their emotional and financial support.

*“If a pregnant woman has a history of convulsion and her husband is aware of her condition her husband could find a person that would be assisting his wife, he would tell the person that “in case my wife convulses, please you would help me assist her” and when they see that the pregnant woman is heavy and she’s about to deliver they shouldn’t let her be alone, they shouldn’t allow her to be alone.because it could affect her pregnancy.” [New Mother]*

In addition to the role of men, diet was perceived as a significant moderator of high blood pressure. Decreased intake of ‘maggi’ – a common food seasoning - and salt were thought to be appropriate methods for preventing high blood pressure among pregnant women. Other prevention strategies included a public health enlightenment programme on preventive methods of pre-eclampsia and its management at the community level. In addition, the roles of community health workers in expanding knowledge and creating awareness on pre-eclampsia and eclampsia conditions was perceived to be very crucial.

### Outcomes of pre-eclampsia and eclampsia

There was a general perception that pre-eclampsia is severe and could lead to loss of life in pregnancy. This was reinforced by individual experiences of pre-eclampsia. One male decision-maker gave a description of high blood pressure in pregnancy as *“a trap”*.

*“High blood pressure is like a trap, once it affects a woman, it may be difficult to cure. Sometime it may be hereditary and once a woman has it, she can also transfer it to her unborn baby since they both share the same blood, high blood pressure can lead to the death of women.” [Male Decision-Maker]*

Furthermore, a view expressed among a segment of the population showed that “*high blood pressure can affect the brain [and] it can also lead to “gìrì” (convulsions), or “òyì orí” (dizziness).* The term *“òyì orí”* combines two Yoruba words; “*òyì”* literally means *dizziness, while “orí”* refers to *head.* Thus, *“òyì orí”* signifies dizziness in the head which could lead to a fatal fall. TBAs described convulsions in pregnancy as a complicated condition. One TBA explained that “*gìrì ipá” (convulsions)* could lead to *“âbísíwín”*, known as puerperal psychosis in biomedical terms. According to new mothers, possible outcomes of pre-eclampsia and eclampsia were premature birth, stillbirth, paralysis, and stroke.

### Traditional treatment of pre-eclampsia and eclampsia

Home-based and traditional treatments for pregnancy complications were very common in the region. There were many traditional treatments used for eclampsia including eating onion, drinking salt solution, and applying Robb to the chest. Robb is a type of balm used for relieving aches and pains among children and adults in addition to its use for treating cold and shivering condition. Other traditional treatment options mentioned were bodily incisions and prayers. According to one woman, “*they could give the pregnant woman onions, they should shred the onions and put in it in her mouth, the aroma of the onions would calm her down, before they take her to the hospital”.* As eclampsia was believed to be caused by exposure to cold, a concoction known as “*oògùn ilè tútù”* meaning *cold ground medicine* was widely reported as one of the traditional medicines used*.* The role of cold-ground-medicine is highlighted in the following quote by a pregnant woman:

*“If it (convulsion) happens, the woman would be given a local concoction “oògùn ilè tútù” or would be taken to the hospital. But she would usually be given the local concoction until the convulsion subsides.” [Pregnant Woman]*

In the treatment of eclampsia, some put salt in the pregnant woman’s mouth as it is believed “*salt would dissolve the substance blocking the blood vessels”.* In addition to giving salt, a spoon or ‘*chewing stick’* is often inserted into the mouth to avoid clenching the teeth. Other types of treatment include asking the woman to lie down on her right side, and then pouring water on her head until she is revived.

*“They should turn her to her right side and raise her up…that is what I know and that is what I’ve witnessed, if they pour water on her head and raise her up and put her down gently. Nobody should say a word around her at that moment. If it’s just a normal convulsion, she would be revived.” [New Mother]*

Lime and hot spices were also reportedly used for treating eclampsia. A TBA described the contents of one of the local concoctions used for eclampsia:

*“I use original tobacco leaves… use it with boiling water and soak it with lemon juice, if pregnant woman is convulsing give her one teaspoon, rub it on her eyes and body… it will usually calm her down.” [Traditional Birth Attendant*]

Some opinion leaders reported the use of incisions on the forehead or abdomen of pregnant women for treating eclampsia. This was usually combined with other local items such as black soap, concoctions, and burnt leaves.

*“They treat it in the traditional way, using [herbs], black soap, burnt leaves or make incisions. You would see some pregnant women with many incisions on their heads.” [Community Leader*]

Among those who patronized prayer houses and spiritualists, different treatment methods were reported: *holy water*. In addition, a special prayer session was reportedly carried out for these women.

## Discussion

The findings of this study show that community perceptions of pre-eclampsia and eclampsia in Ogun State differ significantly from biomedical perspectives [9]. While the perceived cause of eclampsia in these communities was exposure to cold, biomedical evidence suggests that the causal pathway is related to abnormal placentation [14]. Divergent community perspectives reported in this study have led to treatment in line with traditional norms and values in Ogun State, Nigeria. Local treatments in these communities have stemmed from the community’s understanding of the condition.

In addition, the findings show that respondents do not use specific terms for pre-eclampsia in local language, though hypertension has names that independent of pregnancy status that are used. These results demonstrate limited knowledge and awareness of pre-eclampsia and its associated consequences despite its rating as one of the leading causes of maternal death [1, 2, 15, 16]. There was consensus amongst the community on the causes of hypertension, both during and outside pregnancy, with the main cause pointing to psychological factors, particularly depressive thoughts, heredity and stress. There was a similar consensus regarding the cause of eclampsia, the origin reportedly related to an exposure to cold. These findings further show that a knowledge gap exists regarding the causes and progression of symptoms from pre-eclampsia to eclampsia. This lack of awareness underscores the need for a sustained commitment to community sensitization using a combination of approaches that could penetrate social and cultural norms.

There is need for further sensitization of the community members with respect to availability of subsidized health services. The process for sensitization also offers opportunity for education regarding pre-eclampsia and eclampsia. There is a need to promote a strategy that allows community members to intervene using cultural norms to address challenges in care delivery for women with pre-eclampsia and eclampsia. In this sense, this study advocates for a “*cultural intervention”*. As distinct from other forms of intervention, cultural intervention will enable the community to discuss their problems and challenges with the aim of providing solutions to them. The involvement of men in maternal health could be culturally influenced. The role of men in pregnancy and delivery care among African women has gained attention recently, [17, 18] and could be extended to issues related to pre-eclampsia and eclampsia.

This study reveals that there are several local treatments for eclampsia among community members in Ogun state. The use of traditional medicine during pregnancy has been documented elsewhere [19]. The desire to have personal control over their health, dissatisfaction with conventional treatments, and concerns about the side effects of medications explain in part the use of herbal remedies during pregnancy [20]. The efficacy of local interventions, especially those related to the treatment of eclampsia is either not known or deleterious (i.e. placing items between the teeth). The use of traditional treatment has been identified as a source of delay to accessing appropriate health care services and also could be a marker for harmful behaviour [21]. The findings from this study provide additional information on local perception and practices that have consequences for the early treatment of pre-eclampsia and eclampsia in Nigeria.

The general perception that psychological distress could be an underlying cause of pre-eclampsia and eclampsia requires further exploration. This study shows that there is a large gap between community perceptions of pre-eclampsia and eclampsia and the biomedical perspective. Sadly, relevant policies, like the National Health Policy in Nigeria, do not take into account community perspectives in framing such policies. As such, these policies are generally disconnected from peoples’ experiences, beliefs and local realities.

### Strengths and limitations

One of the strengths of this study is the community-based approach, which provided rich insight into perceptions of the diverse population. In addition, the participation of the various stakeholder groups who influence beliefs and attitudes enriched the study findings: pregnant women, opinion leaders, religious leaders, TBAs and male decision-makers. Furthermore, a multi-disciplinary approach brought researchers from various backgrounds to enhance quality in data collection. Notwithstanding, findings from this study may not apply to other ethnic groups represented in Nigeria other than the Yoruba among whom the study was conducted. Another limitation is the inability of the study to provide in details regarding the contents of local medicines in use during pregnancy.

## Conclusions

The study illustrates that knowledge of pre-eclampsia and eclampsia are limited amongst communities of Ogun State, Nigeria; there are gaps in knowledge regarding the aetiology and treatment of the conditions. It also highlights the need for a review of maternal health policies in Nigeria with special attention to community roles, specifically the role of men, and the need for health care providers to be equipped with appropriate skills and relevant materials to provide community education and sensitization to improve maternal and perinatal health.

## Additional file

Additional file 1: Peer review reports. (PDF 274 kb)

## Abbreviations

HDP: Hypertensive disorders of pregnancy
LGA: Local Government Areas
TBA: traditional birth attendants
