# Supplementary material for: Community perceptions of pre-eclampsia and eclampsia in Ogun State, Nigeria: a qualitative study
Source: Reprod Health. 2016 Jun 8;13(Suppl 1):57. doi: 10.1186/s12978-016-0134-z (PMC4943493; doi:10.1186/s12978-016-0134-z)
Supplement: Additional file 1: — Peer review reports. (PDF 274 kb) [file 12978_2016_134_MOESM1_ESM.pdf]

Referee's comments to the authors– this sheet WILL be seen by the author(s) and published with the article

Title	**Community perceptions of pre-eclampsia and eclampsia in Ogun State, Nigeria: A qualitative study**
Author(s)	David O Akeju, Marianne Vidler, Olufemi T. Oladapo, Diane Sawchuck, Rahat Qureshi, Peter von Dadelszen, Olalekan O. Adetoro, Olukayode A. Dada andd the CLIP Nigeria Feasibility Working Group
Referee's name	Sanjay Gupte

When assessing the work, please consider the following points, where applicable:

1. Is the question posed by the authors new and well defined? Yes
2. Are the methods appropriate and well described, and are sufficient details provided to replicate the work? Yes
3. Are the data sound and well controlled? Yes
4. Does the manuscript adhere to the relevant standards for reporting and data deposition? Yes
5. Are the discussion and conclusions well balanced and adequately supported by the data? Yes
6. Do the title and abstract accurately convey what has been found? Yes
7. Is the writing acceptable? Yes

Please make your report as constructive and detailed as possible in your comments so that authors have the opportunity to overcome any serious deficiencies that you find and please also divide your comments into the following categories:

- Major Compulsory Revisions (which the author must respond to before a decision on publication can be reached)
- Minor Essential Revisions (such as missing labels on figures, or the wrong use of a term, which the author can be trusted to correct)
- Discretionary Revisions (which are recommendations for improvement but which the author can choose to ignore)

Where possible please supply references to substantiate your comments.

When referring to the manuscript please provide specific page and paragraph citations where appropriate.

General comments:
Innovative & interesting study

Major compulsory revisions:

Minor essential revisions:

Discretionary revisions:

(continue on the next sheet)

Referee's comments to the authors– this sheet WILL be seen by the author(s) and published with the article

Title	**Community perceptions of pre-eclampsia and eclampsia in Ogun State, Nigeria: A qualitative study**
Author(s)	David O Akeju, Marianne Vidler, Olufemi T. Oladapo, Diane Sawchuck, Rahat Qureshi, Peter von Dadelszen, Olalekan O. Adetoro, Olukayode A. Dada andd the CLIP Nigeria Feasibility Working Group
Referee's name	Tessa Gillon

When assessing the work, please consider the following points, where applicable:

1. Is the question posed by the authors new and well defined?
2. Are the methods appropriate and well described, and are sufficient details provided to replicate the work?
3. Are the data sound and well controlled?
4. Does the manuscript adhere to the relevant standards for reporting and data deposition?
5. Are the discussion and conclusions well balanced and adequately supported by the data?
6. Do the title and abstract accurately convey what has been found?
7. Is the writing acceptable?

Please make your report as constructive and detailed as possible in your comments so that authors have the opportunity to overcome any serious deficiencies that you find and please also divide your comments into the following categories:

- Major Compulsory Revisions (which the author must respond to before a decision on publication can be reached)
- Minor Essential Revisions (such as missing labels on figures, or the wrong use of a term, which the author can be trusted to correct)
- Discretionary Revisions (which are recommendations for improvement but which the author can choose to ignore)

Where possible please supply references to substantiate your comments.

When referring to the manuscript please provide specific page and paragraph citations where appropriate.

General comments:
It seems like the coding structure has been well thought about by the authors FO & OA. I think the authors are right to have had the coding done by a single author (MV) in order to preserve consistency. However, It might have been interesting for two authors to perform coding individually which would have allowed for comparison and the resolution of inconsistencies, should that emerge. I realize this suggestion requires additional work, and may not be suitable or necessary for all qualitative studies. However, having different points of view to examine can be, in my opinion, of added value.

I believe this study gives us another angle to a problem widely investigated in developed countries. Change for better care in low in mid-income countries is only possible if people's perceptions are understood. This study shows that some basic perceptions or thinking processes of the researched community are headed in the right direction (e.g. Lines 165-172: association between high BP and "a substance in her urine" and swollen legs). But the lack of biomedical knowledge clearly leads to "false" perceptions and insufficient management of the diseases.

In my opinion the title and abstract do convey very well what has been concluded.
The writing is clear and understandable.

Major compulsory revisions:
None

Minor essential revisions:
None

Continued:

Discretionary revisions:
On page 10 Line 187-189, it is mentioned that giving access to knowledge that could aid in the prevention of PE and eclampsia is part of the prevention strategy. Could you add examples of what that knowledge is and how access is given?

I would be interested to see the questions asked in the focus group discussions. I suggest that the main questions/discussion subjects be added as an attachment, which might lead to better insights on the thought processes of the locals which led to some of the conclusions arrived at.

Supplement Editor's comments

I wonder if in the conclusion of the abstract instead of mentioning:
"This study illustrates that knowledge of pre-eclampsia and eclampsia are limited amongst 24 communities of Ogun State, Nigeria. Furthermore, findings reveal the existence of gap in 25 knowledge regarding the aetiology and treatment of the conditions."
You can briefly describe which are the major constraints in knowledge and the gaps.

Declaration of competing interests
We ask all peer reviewers to declare their competing interests in relation to the paper they are reviewing. The peer reviewer declaration is included in the confidential comment report.

In the context of peer review, a competing interest exists when your interpretation of data or presentation of information may be influenced by your personal or financial relationship with other people or organizations. Reviewers should disclose any financial competing interests but also any non-financial competing interests that may cause them embarrassment were they to become public after the publication of the manuscript.

When completing your declaration, please consider the following questions:

- Have you in the past five years received reimbursements, fees, funding, or salary from an organisation that may in any way gain or lose financially from the publication of this paper, either now or in the future?
- Do you hold any stocks or shares in an organisation that may in any way gain or lose financially from the publication of this paper, either now or in the future?
- Do you hold or are you currently applying for any patents relating to the content of the manuscript? Have you received reimbursements, fees, funding, or salary from an organization that holds or has applied for patents relating to the content of the manuscript?
- Do you have any other financial competing interests?
- Do you have any non-financial competing interests in relation to this paper?

If you can answer no to all of the above, write 'I declare that I have no competing interests' in the space on page 1. If your reply is yes to any, please give details on page 1.
